# Giant concha bullosa presented as left nasal mass: a case report and literature review

**DOI:** 10.1093/jscr/rjad558

**Published:** 2023-10-17

**Authors:** Feras M Al-Kholaiwi, Reema A Al-Khatabi, Ghada A Al-Shehri, Yazeed A Al-Ghonaim

**Affiliations:** Department of Otolaryngology - Head and Neck Surgery, Imam Mohammad Ibn Saud Islamic University (IMSIU) 11564, Riyadh, Saudi Arabia; College of Medicine, Imam Mohammad Ibn Saud Islamic University (IMSIU) 11564, Riyadh, Saudi Arabia; College of Medicine, Imam Mohammad Ibn Saud Islamic University (IMSIU) 11564, Riyadh, Saudi Arabia; Department of Otolaryngology - Head and Neck Surgery, King Saud Bin Abdulaziz University for Health Sciences (KSAU-HS) 11481, Riyadh, Saudi Arabia

**Keywords:** unilateral nasal mass, middle turbinate, nasal obstruction, concha bullosa, rhinology

## Abstract

Concha bullosa (CB) is not considered to be a disease, but rather a variation of the paranasal sinus. A CB is defined as the presence of an air cell within the turbinate (pneumatization). The main function of CB is to maintain upper respiratory humidity, regulate thermoregulation, and regulate airflow and filtration. It is common for CB to occur in the middle turbinate, while superior and inferior locations appear to be rare. The patient in this case report was presented mainly with nasal obstruction. During examination, a large mass was found in the left nasal cavity, causing a deviated nasal septum to the right. For this patient, surgery was the last resort. Either perioperative or postoperative complications were observed, and the quality of life of patients improved after surgery.

## Introduction

Concha bullosa (CB) is not described as a disease itself it is consider under the variation of the paranasal sinuses [1, 2]. Concha bullosa is described when the ethmoidal air cell is present inside the turbinate (pneumatization) [1]. The middle turbinate found to be the one of the most common of all the paranasal anatomical variation pneumatization with incidence ranges from 14 to 53% [3], in the other hand the superior and inferior found to be extremely infrequent [1, 2]. Concha bullosa could be unilateral or bilateral [1]. Patients are usually asymptomatic, unless they have hyper-pneumatization, and they will present with symptoms, such as nasal obstruction, headache, and chronic sinusitis [1, 3]. Nevertheless, any disease process of the paranasal sinus can impact the CB resulting in mucocele, pyocele, and mucocele thickening [4, 5]. However, the outcomes of this are still infrequent. In this article, we describe a rare huge unilateral middle pneumatization that refractory to medical treatment in over a year duration in patient with significant symptoms.

## Case report

**Figure 1:**
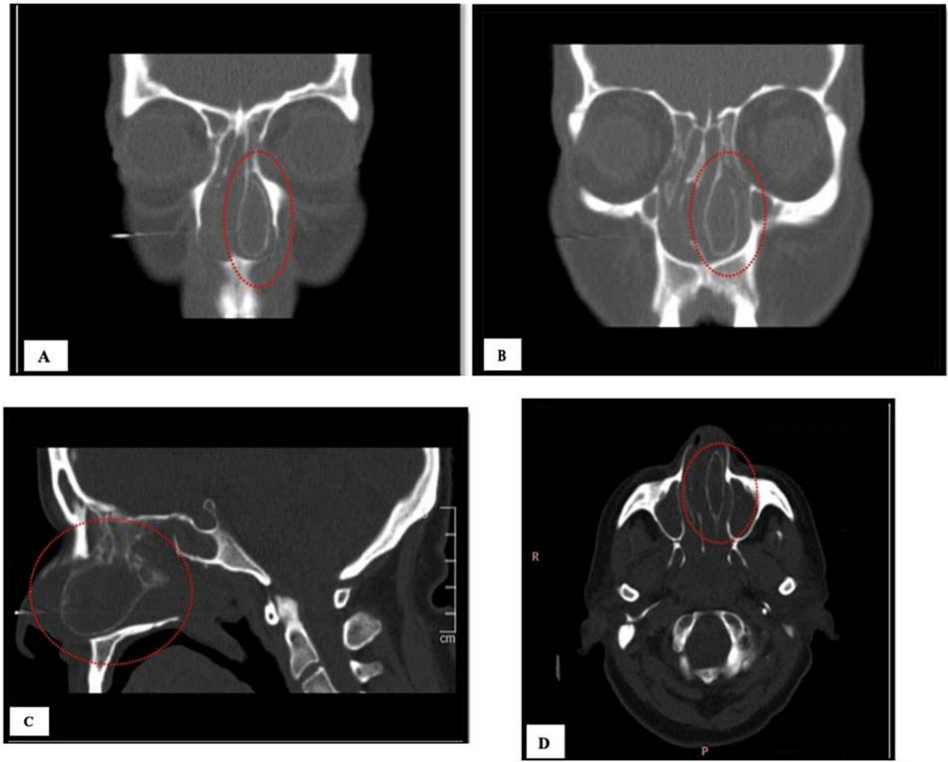
(A, B) CT of coronal view; (C) CT of sagittal view, and (D) CT of axial view.

**Figure 2:**
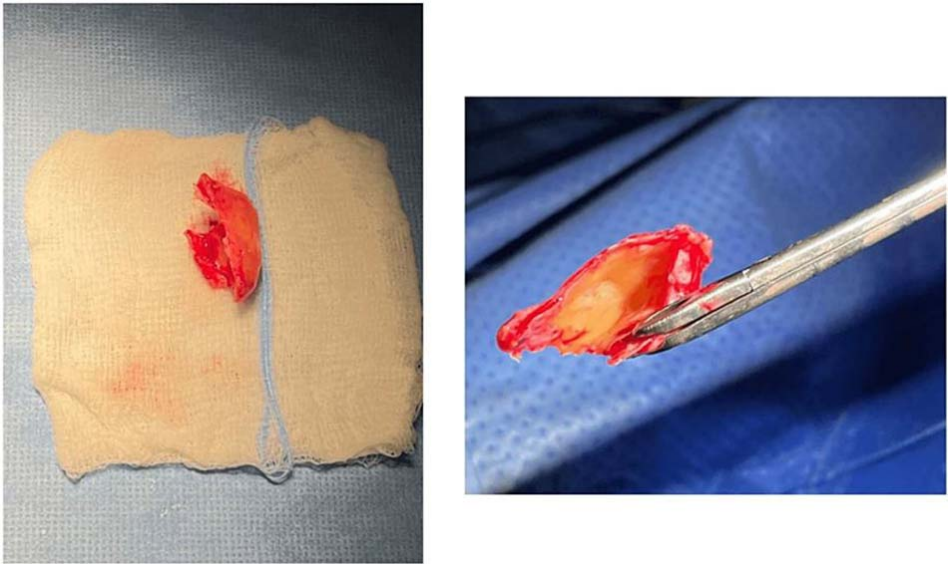
The giant concha bullosa after resection, which was obstructing the left nasal cavity.

A 64-year-old female presented to our clinic complaining of nasal obstruction more to left side, postnasal discharge, and intermittent headache, anosmia, and facial discomfort since 1 year ago. She had sought previous medical advice and was on intranasal steroid spray and nasal saline irrigation without any improvement, her symptoms affecting the quality of life. She is not known history of allergy; she is not a smoker. She does not have any significant past medical or surgical history. On examination, anterior rhinoscopy showed: left nasal cavity huge mass reaching to left nostril and deviated nasal septum to the right. The endoscopic exam showed huge left-sided nasal mass reaching the level of left nostril, the scope cannot be passed to left-sided nasal cavity, right-sided endoscopic examination showed deviated nasal septum, discharge was appreciated from right middle meatus and along with edematous middle meatus. The nasopharyngeal examination showed post-nasal discharge over the wall without any mass. Rest of complete ears, throat, neck, and cranial nerves examinations were normal. The patient underwent head and paranasal sinus computed tomography (CT) scan, and it shows that this giant CB was pushing the septum to right, as well obstructing sinuses regular drainage contributing to the chronic sinusitis symptoms (Fig. 1). The patient was admitted to hospital for functional endoscopic sinus surgery, left CB release, excision, and septoplasty (Fig. 2). The specimen was collected in a formalin and send to further evaluation and came confirming the diagnosis with “polypoid fragment of respiratory mucosa with cystic space and inflammation,” and it was negative for any malignancy. No perioperative or postoperative complications were seen. The post-operative period showed excellent improvement in patient symptoms. Her quality of life much improved, she is on regular follow up in the clinic along regular nasal endoscopic examination with no evidence of disease recurrence till 12 months post-surgical intervention.

## Discussion

Concha bullosa is not a disease in itself, but rather a variation of the paranasal sinuses [1, 2]. The cause it still unknown but could be related to anatomical septum deviation or to the aeration of middle turbinate [2, 3]. Concha bullosa occurs when ethmoidal air cells are present inside the turbinate (pneumatization) classified into three groups: lamellar CB, bulbous CB, and extensive CB [2]. However, the middle concha pneumatizations is an extension of normal pneumatization of ethmoid air cells. About 55% and 45% of all the cases of the pneumatization of the middle turbinate (PMT) are liable by the anterior ethmoid cell from the middle meatus and the posterior ethmoid cells from the superior meatus, respectively [2, 3]. Concha bullosa has a variety of anatomical variations and could present as vertically combined double, septated CB, paradoxical curvature of middle turbinate, ipsilateral paradoxical superior turbinate, contralateral paradoxical middle turbinate, CB superior, or CB suprema [6]. Concha bullosa is commonly found incidentally and asymptomatic [3, 7]. Patients with extensive bullous concha will be symptomatic, while patients with bullous and lamellar concha are usually asymptomatic [2]. The amount of pneumatization and inflammation in CB may be correlated with symptoms of the adjacent structures [8]. Symptoms include nasal obstruction, headaches, and smell disturbances [4]. As a result of the inflammation process it may lead to hypertrophy and even polyp formation [4]. Concha bullosa has been linked with an increased risk of chronic sinusitis recurrence due to its negative impact on ventilation of paranasal sinuses and mucociliary clearance around the middle meatus [4] with an estimated prevalence of 8–60% [9]. The diagnosis mainly done with nasal diagnostic endoscopy and paranasal CT [2]. It has been found that the degree of pneumatization is positively associated with the severity of symptoms. Surgical intervention is the most commonly used treatment for symptomatic CB [2]. In asymptomatic patients, surgery is not indicated, and CB is usually not severe enough to require treatment [2]. A surgical approach with various techniques and approaches is a combination of endoscopic partial resection, turbinoplasty, total resection, crushing, and intrinsic stripping in symptomatic patients. Surgical resection of the middle concha lateral lamella is the most commonly used procedure [2]. For CB that is symptomatic, medical trial of antibiotics, topical steroids, antihistamines, and nasal decongestants for short-term symptomatic relief. As this patient used all the listed medical treatment without any improvement prior to the surgery [4]. A few case reports in the literature reported the presence of large CB (Table 1), which caused nasal obstruction and long-term symptoms, such as headaches. All previous case reports showed patients were treated with surgical intervention for giant CB, resulted in complete resolution of symptoms with no postoperative complications [4]. The follow-up varied between studies, with some reporting 1 month follow-up and others extending up to 9 months [4].

**Table 1:** Summary of studies reporting large conchae bullosae.

	Author	Design	Symptoms	Location	Intervention	Complications	Outcomes	Recurrence
1.	Cohen 2008 [13]	Case report	Mild, nonprogressive left nasal congestion, snoring, and two episodes of epi-staxis that had occurred in the past 6 months	Left nasal cavity	Endoscopic resection of a left concha bullosa and anterior ethmoidectomy	NR		NR
2.	Cukurova 2012 [2]	Case report	Nasal breathing difficulties and headache persisting for a long time	Right nasal cavity	The patient underwent resection of the concha bullosa and ethmoidal bulla during ESS, and septoplasty was performed	None	The patient’s headache and nasal obstruction complaints were completely relieved within a short time after surgery	NR
3.	Shihada 2012 [12]	Case report	Recurrent migraine headache	Right nasal cavity	The right middle turbinate was resected, and a right anterior ethmoidectomy was performed	None	No symptoms at 1 month, 6 months, and 12 months post-intervention, no headaches	Nasal endoscopy showed a complete absence of recurrent or residual disease
4.	Derin 2014 [11]	Case report	Long-term nasal obstruction, headache, and frequent attacks of acute sinusitis	Right nasal cavity	The lateral wall of the concha bullosa was excised	No perioperative or postoperative complications were seen	No symptoms, and the nasal passages were clear. Nasal breathing and headache were significantly improved after the surgery, and the patient was free of nasal problems during a 6-month follow-up	None
5.	Sari 2015 [14]	Case report	Increasingly severe and frequent nasal obstruction and headache attacks during the previous week. She did not mention postnasal drip and olfactory impairment	Left nasal cavity	The purulent material was aspirated, and the lateral part of the left turbinate was resected	None	During the following 9 months, the patient developed no additional problems	None
6.	Khalife 2016 [10]	Case series of five patients	Our of which were concha bullosa mucoceles, and one was a mucopyocele. Three of the patients had some form of previous nasal trauma. Headache and nasal obstruction were the most common symptoms, with a nasal mass finding on physical examination	Left (60%), right (40%)	Surgical excision and/or marsupialization in all patients	None	Opening of involved sinuses (100%)	No recurrence in 60% and loss to follow-up in 40%
7.	Fuglsang 2018 [15]	Case report	Concha bullosa pyogenic mucocele. Episodes of migraine. Voice more nasal than usual, snoring, right-sided nasal stenosis, and a constant ipsilateral, serous nasal discharge and effusion from the left ear during the previous 2–3 weeks	Right nasal cavity	Removal of the middle turbinate and the tumor in was performed	None	No nasal symptoms, intermittent effusions from the left ear. At the 6-month follow-up, the mucosa was normalized with no symptoms	None
8.	Al Riyami 2020 [9]	Case report	Left-sided nasal obstruction, headache, and postnasal drip symptoms that have been going on for 2 years	Left nasal cavity	The patient has undergone excision of both concha bullosa as well as a left-sided antrostomy	None	Postoperatively, the patient’s condition remained stable, and symptoms improved	NR

NR, not reported.

## Conclusion

The purpose of this literature review is to provide an overview of the most common clinical presentation and the methods of managing this type of CB.
